# Supplementary material for: SMRT sequencing of the Oryza rufipogon genome reveals the genomic basis of rice adaptation
Source: Commun Biol. 2020 Apr 7;3:167. doi: 10.1038/s42003-020-0890-8 (PMC7138787; doi:10.1038/s42003-020-0890-8)
Supplement: Supplementary file 1 — Supplementary Information [file 42003_2020_890_MOESM1_ESM.pdf]

Supplementary Figures

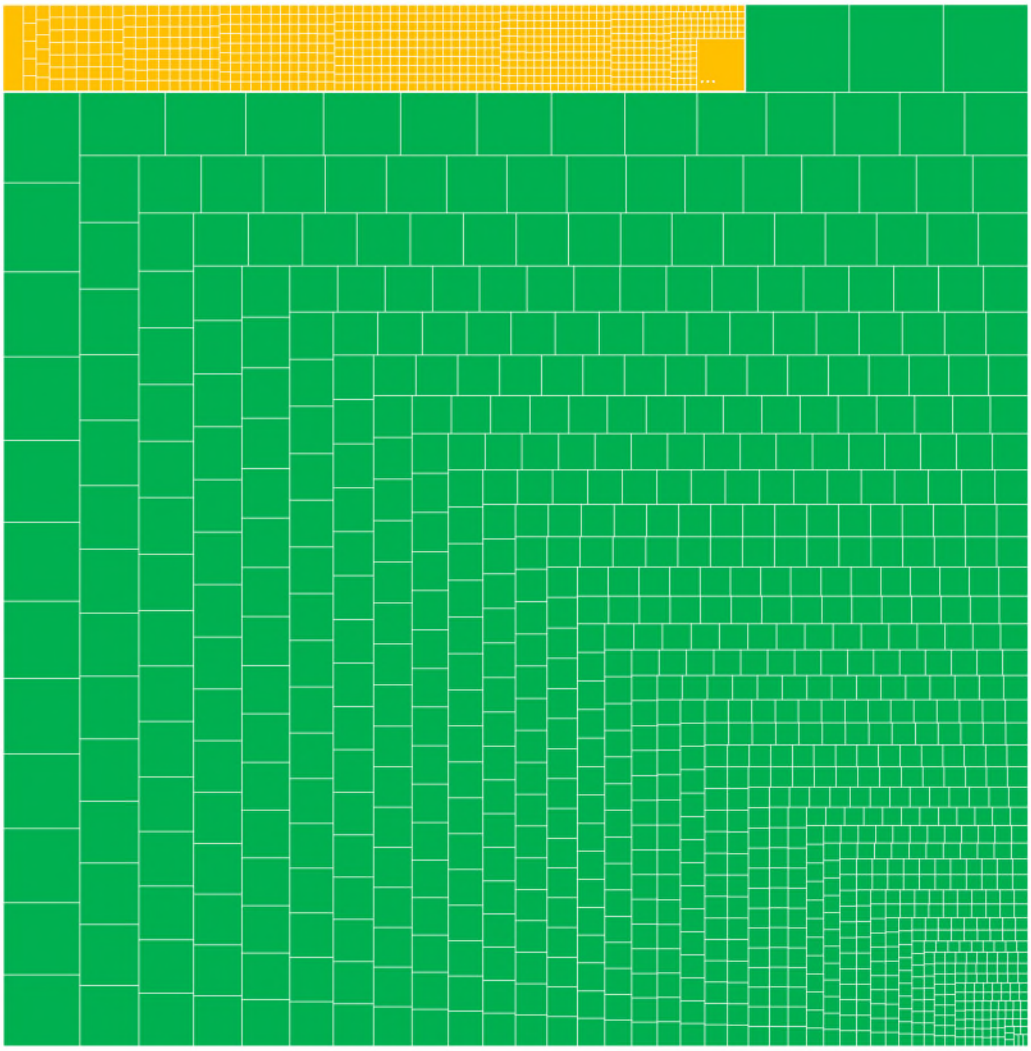

Supplementary Figure 1. Tree map of haplotigs (yellow) and p-contigs (green) scaled by length.

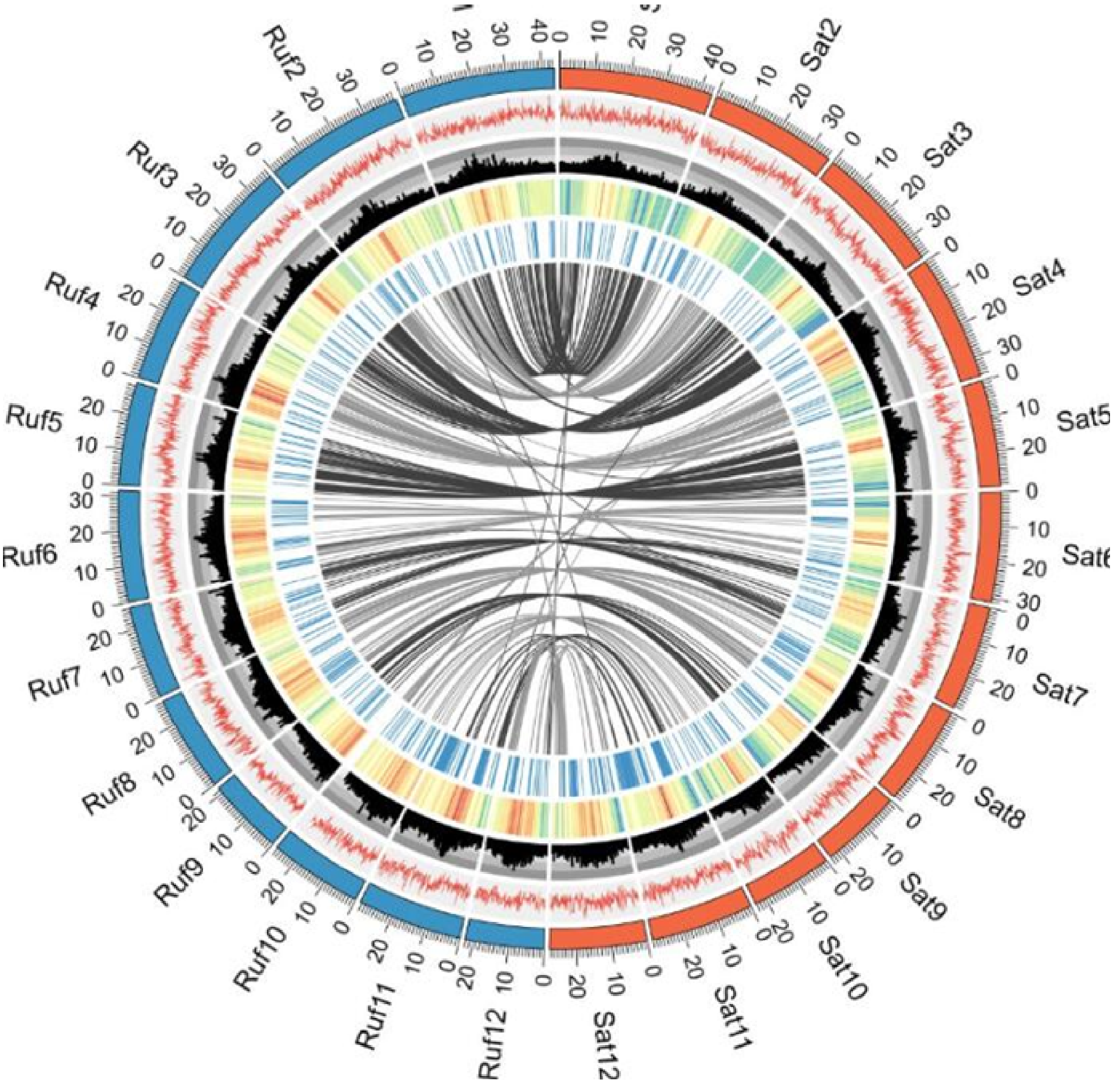

Supplementary Figure 2. **Synteny comparisons and genome features of *O. rufipogon* and *O. sativa***. Tracks from outside to inside are the 12 chromosomes of *O. rufipogon* and *O. sativa*, GC content, transposable element (TE) density, gene density (density measured in 100-Kb sliding windows), and distribution of NBS genes. The syntenic blocks between *O. rufipogon* and *O. sativa* chromosomes are displayed with connecting lines in different colors.

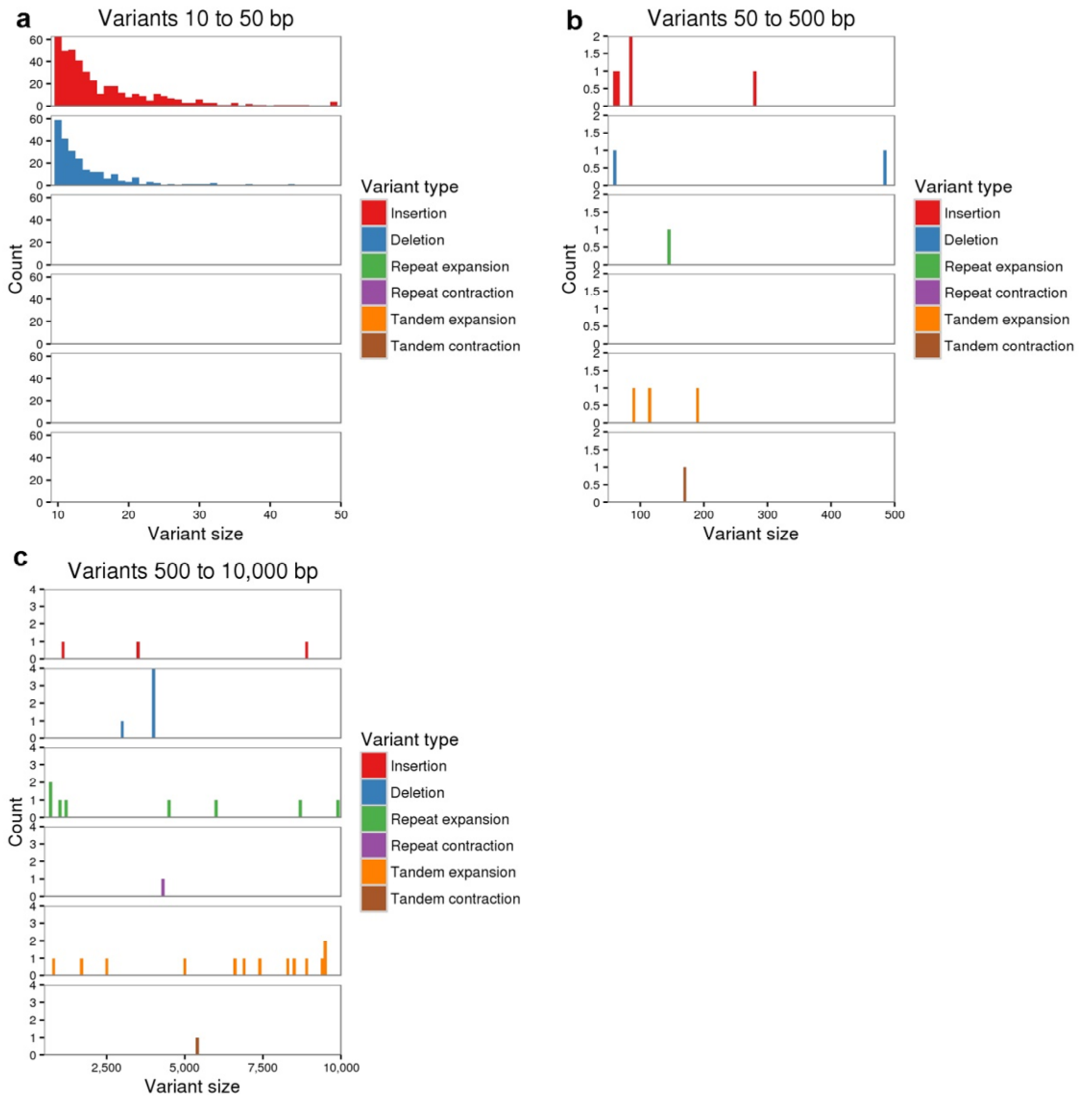

Supplementary Figure 3. Number of genomic variants in different lengths detected between haplotigs and p-contigs. a Variants from 10 to 50 bp; **b** variants from 50 to 500 bp; **c** variants from 500 to 10,000 bp.

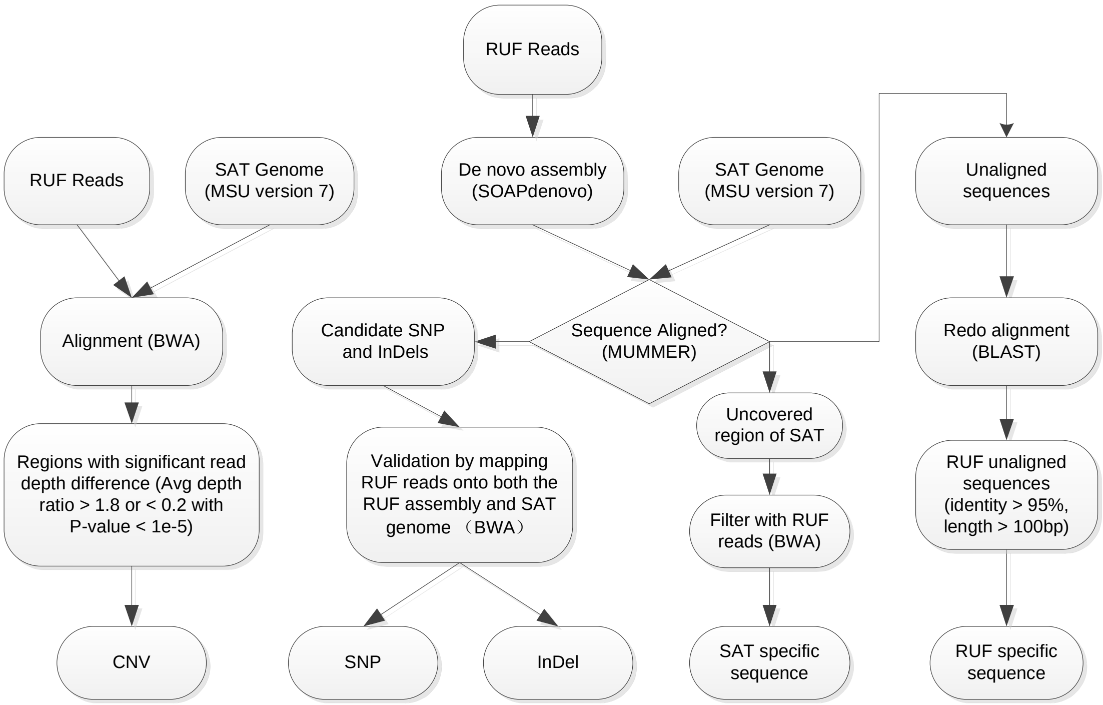

Supplementary Figure 4. Flowchart for the detection of genomic variation between RUF and the Nipponbare genome. This pipeline was also applied to the comparison between the NIV and Nipponbare genomes.

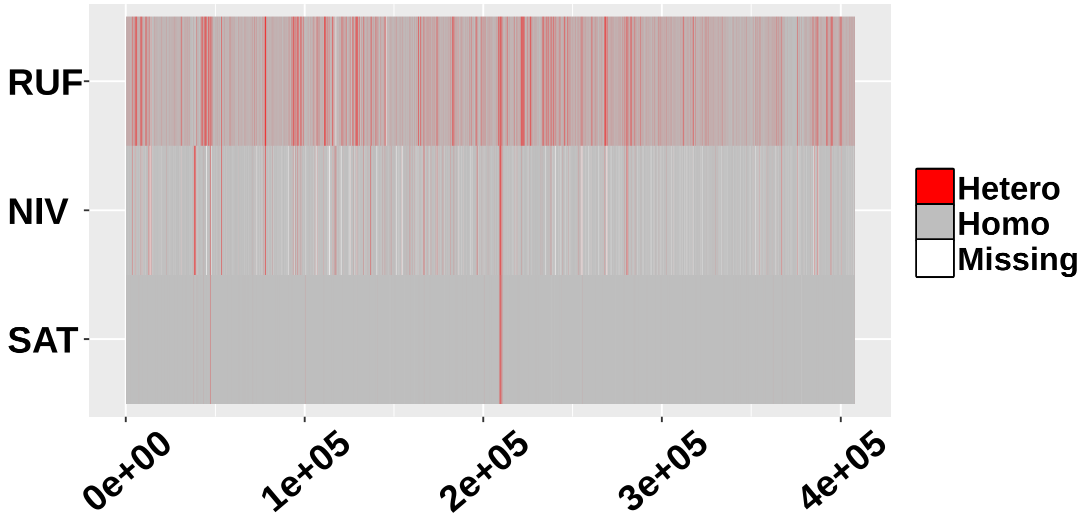

Chromosome 2

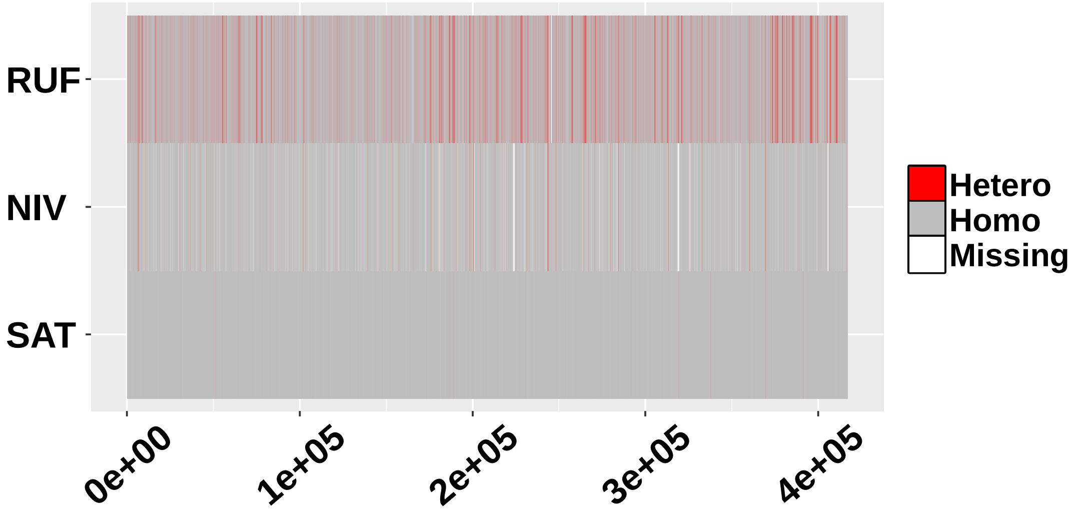

Chromosome 3

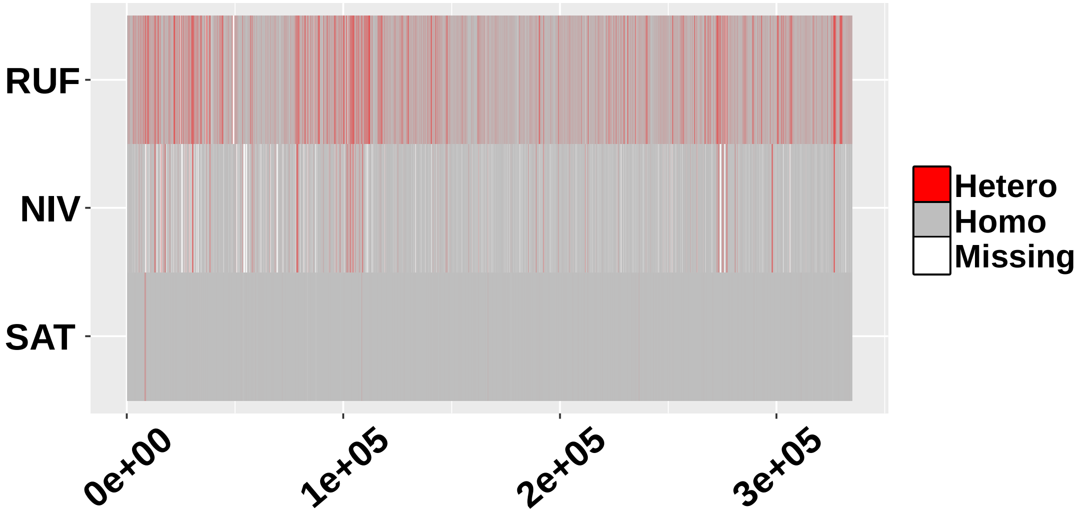

Chromosome 4

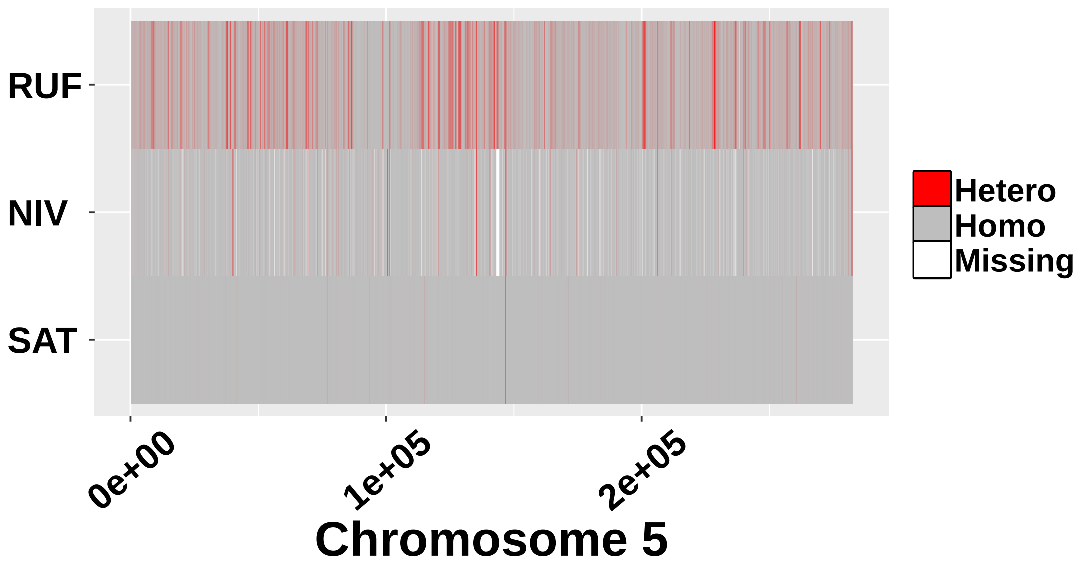
RUF
NIV
SAT
0e+00
1e+05
2e+05
Chromosome 5
Hetero
Homo
Missing

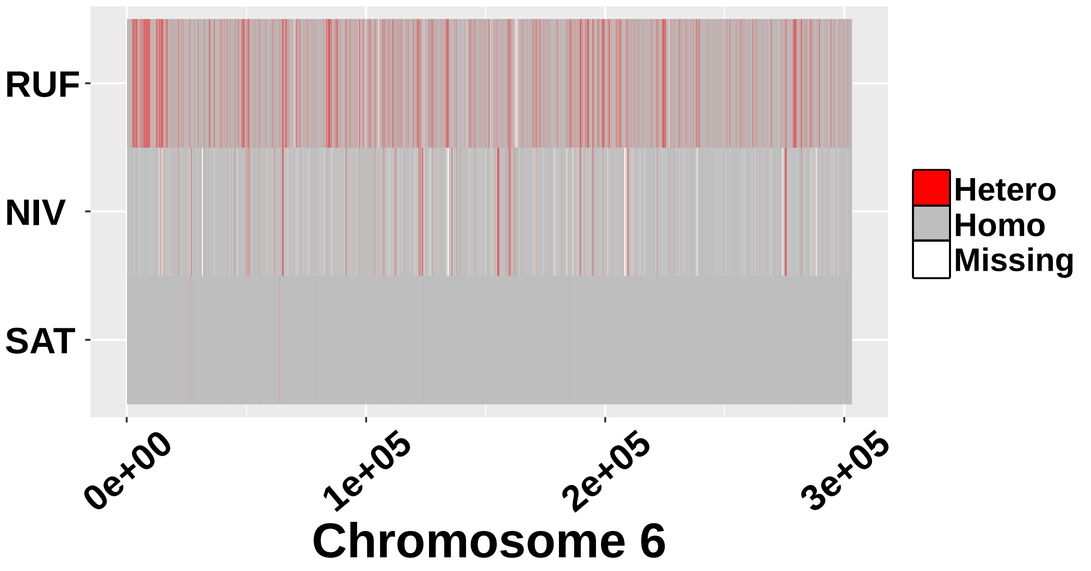
RUF
NIV
SAT
0e+00
1e+05
2e+05
3e+05
Chromosome 6
Hetero
Homo
Missing

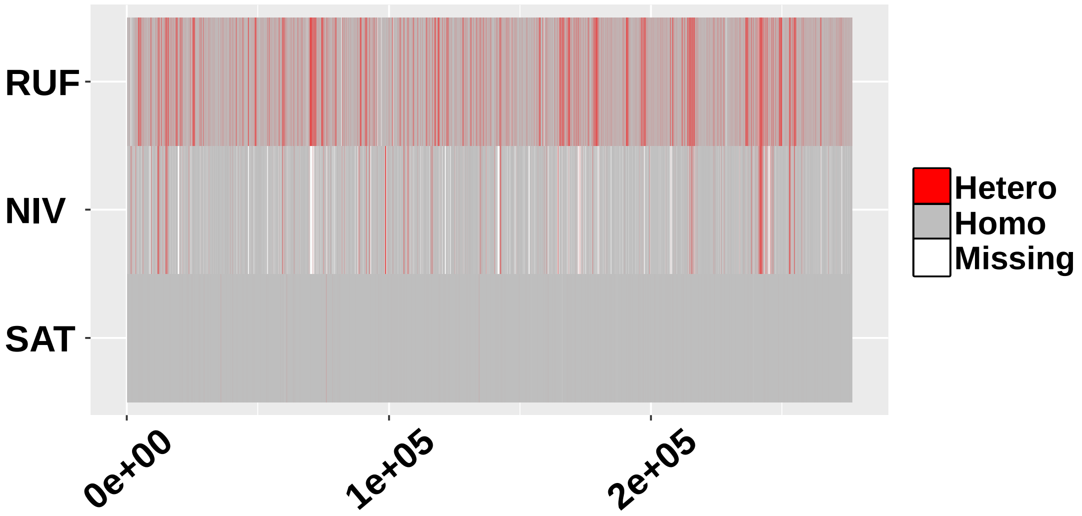

Chromosome 7

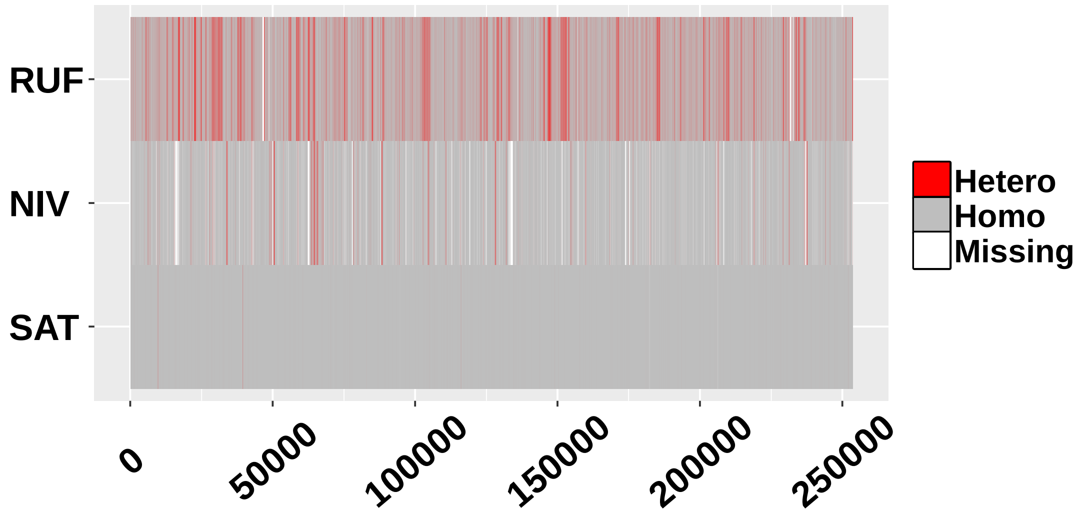

Chromosome 8

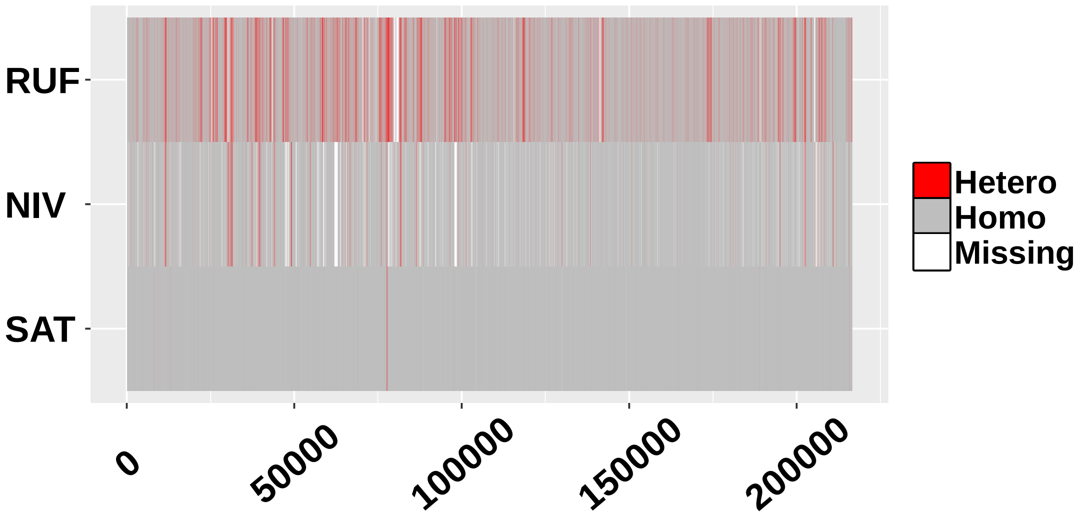

Chromosome 9

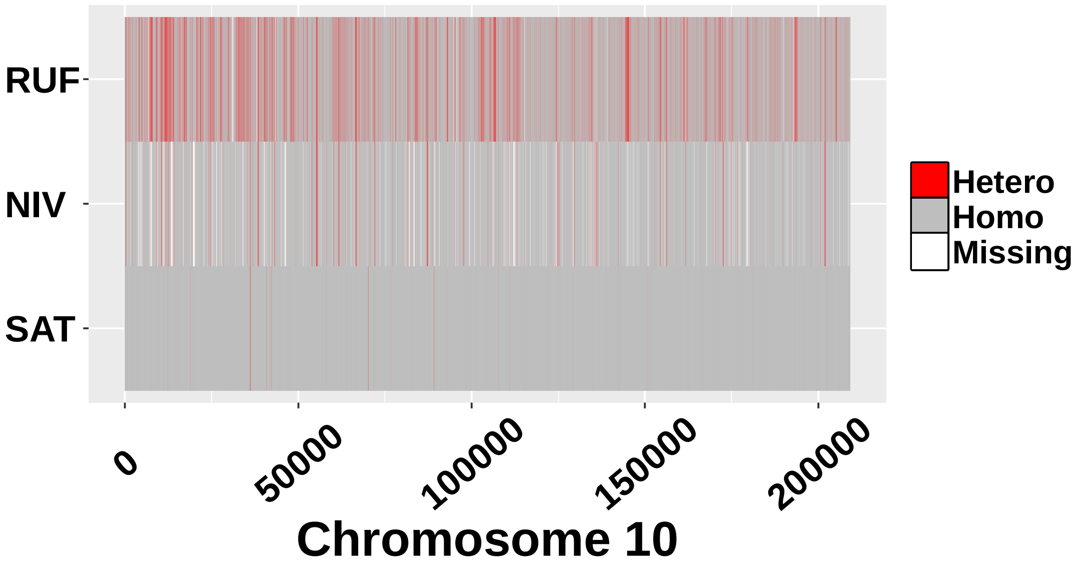
RUF
NIV
SAT
0
50000
100000
150000
200000
Chromosome 10
Hetero
Homo
Missing

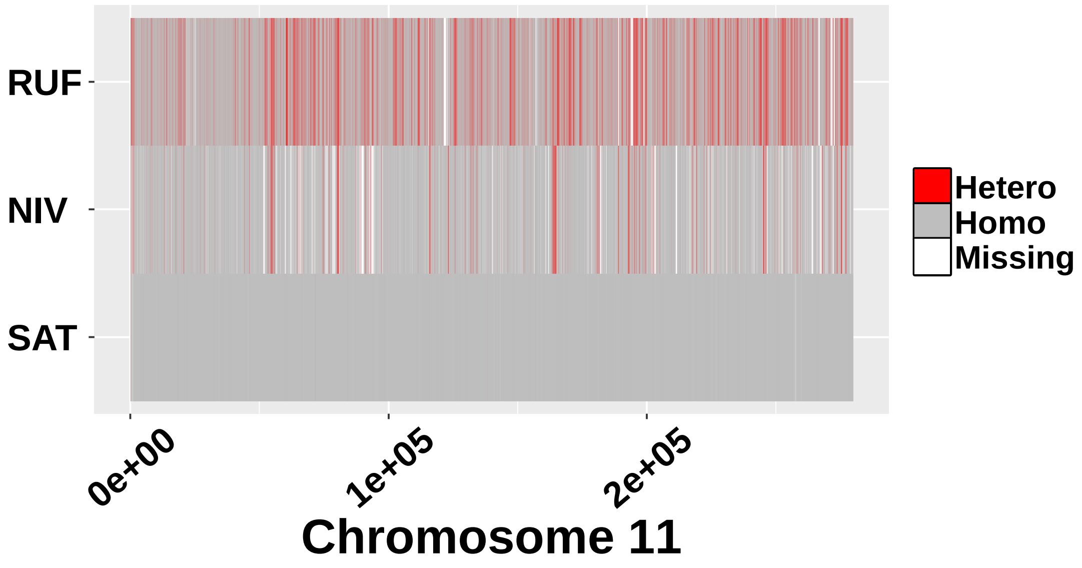
RUF
NIV
SAT
0e+00
1e+05
2e+05
Chromosome 11
Hetero
Homo
Missing

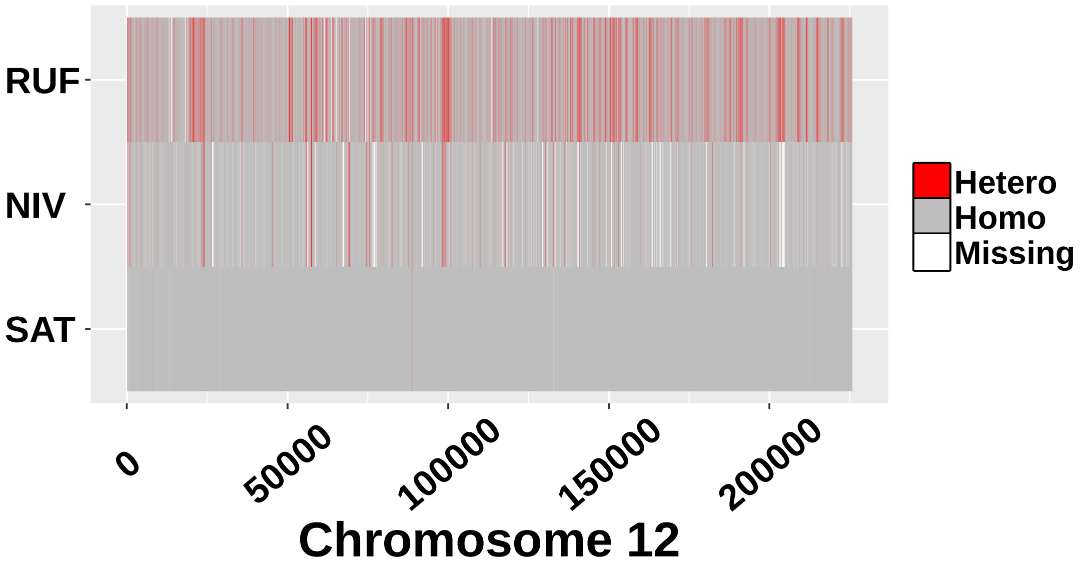
RUF
NIV
SAT
0
50000
100000
150000
200000
Chromosome 12
Hetero
Homo
Missing

Supplementary Figure 5. Patterns of single nucleotide polymorphisms along the 11 rice chromosomes among *O. rufipogon*, *O. nivara* and *O. sativa*. Heterozygous and homozygous SNPs are shown with red and gray lines, respectively, while the unknown sites are indicated with white lines.

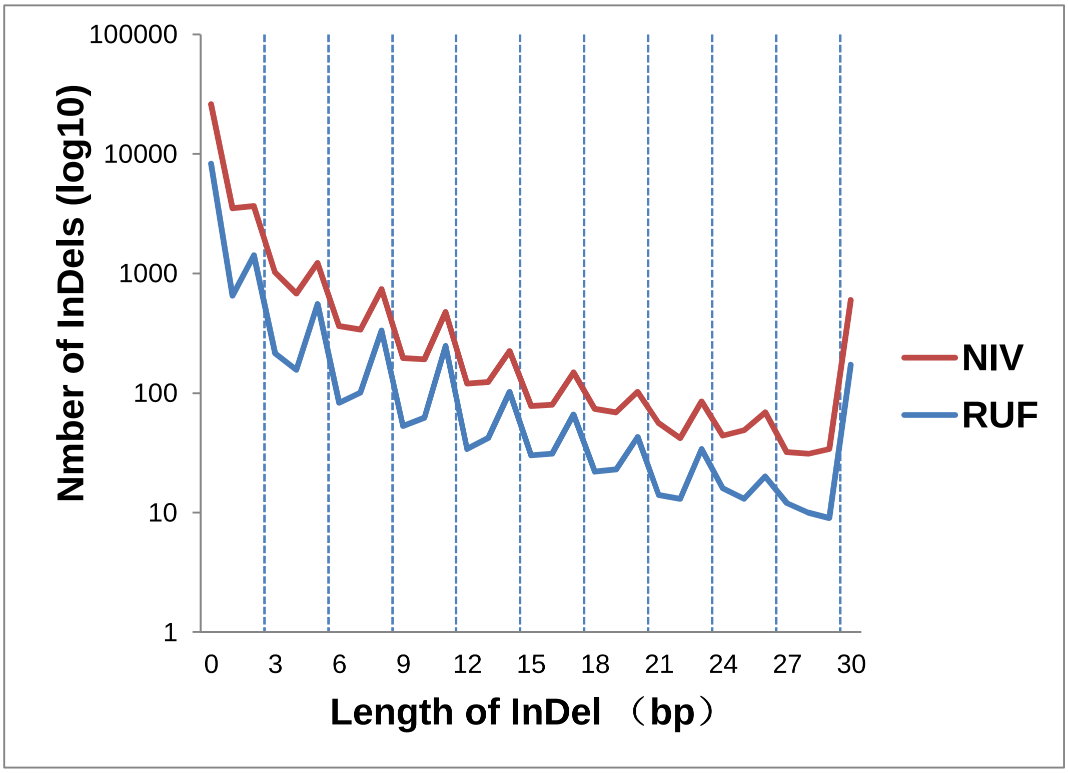

Supplementary Figure 6. Length distribution of InDels located within CDS of *O. rufipogon* and *O. nivara*. The peaks at positions that are multiples of three are shown by dashed vertical lines.

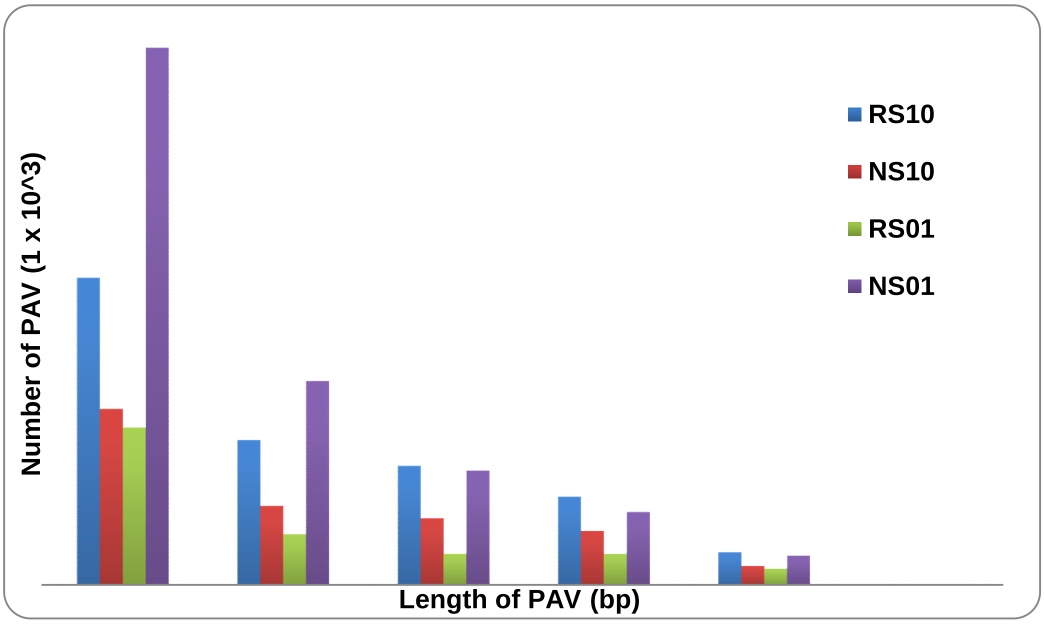

Supplementary Figure 7. Summary of PAVs among *O. rufipogon*, *O. nivara* and *O. sativa*. R: *O. rufipogon* (RUF); S: *O. sativa* (SAT); N: *O. nivara* (NIV); 1: presence; 0: absence. We defined and assigned four types of PAV: RS10 (presence in RUF but absence in SAT), RS01 (presence in SAT but absence in RUF), NS10 (presence in NIV but absence in SAT) and NS01 (presence in SAT but absence in NIV).

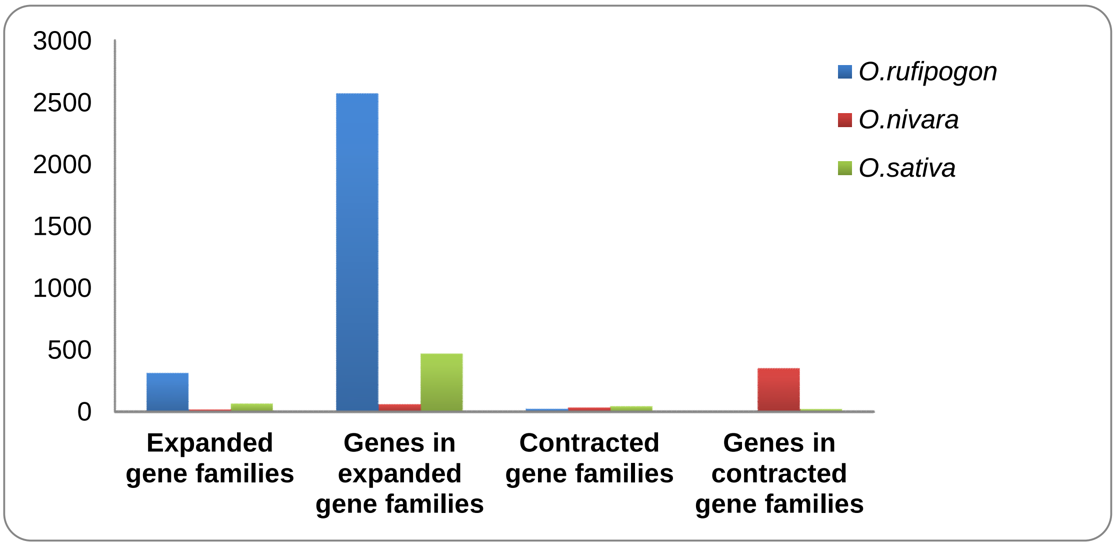

Supplementary Figure 8. Summary of gene families expanded or contracted significantly (*P* < 0.01) in terminal branches of *O. rufipogon*, *O. nivara* and *O. sativa*.

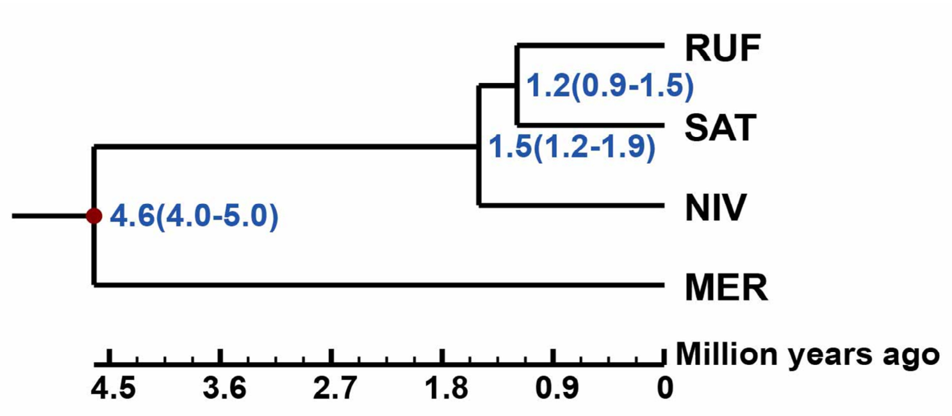

Supplementary Figure 9. Phylogenetic relationships and divergence times of *O. rufipogon* (RUF), *O. nivara* (NIV) and *O. sativa* (SAT) using *O. meridionalis* (MER) as outgroup. The phylogeny was inferred from 10,206 high-confidence 1:1 orthologous gene families based on the maximum likelihood method under the GTR+ GAMMA model, and the divergence times were estimated using *mcmctree* program implemented in PAML. Note that all nodes are fully supported at 100%.

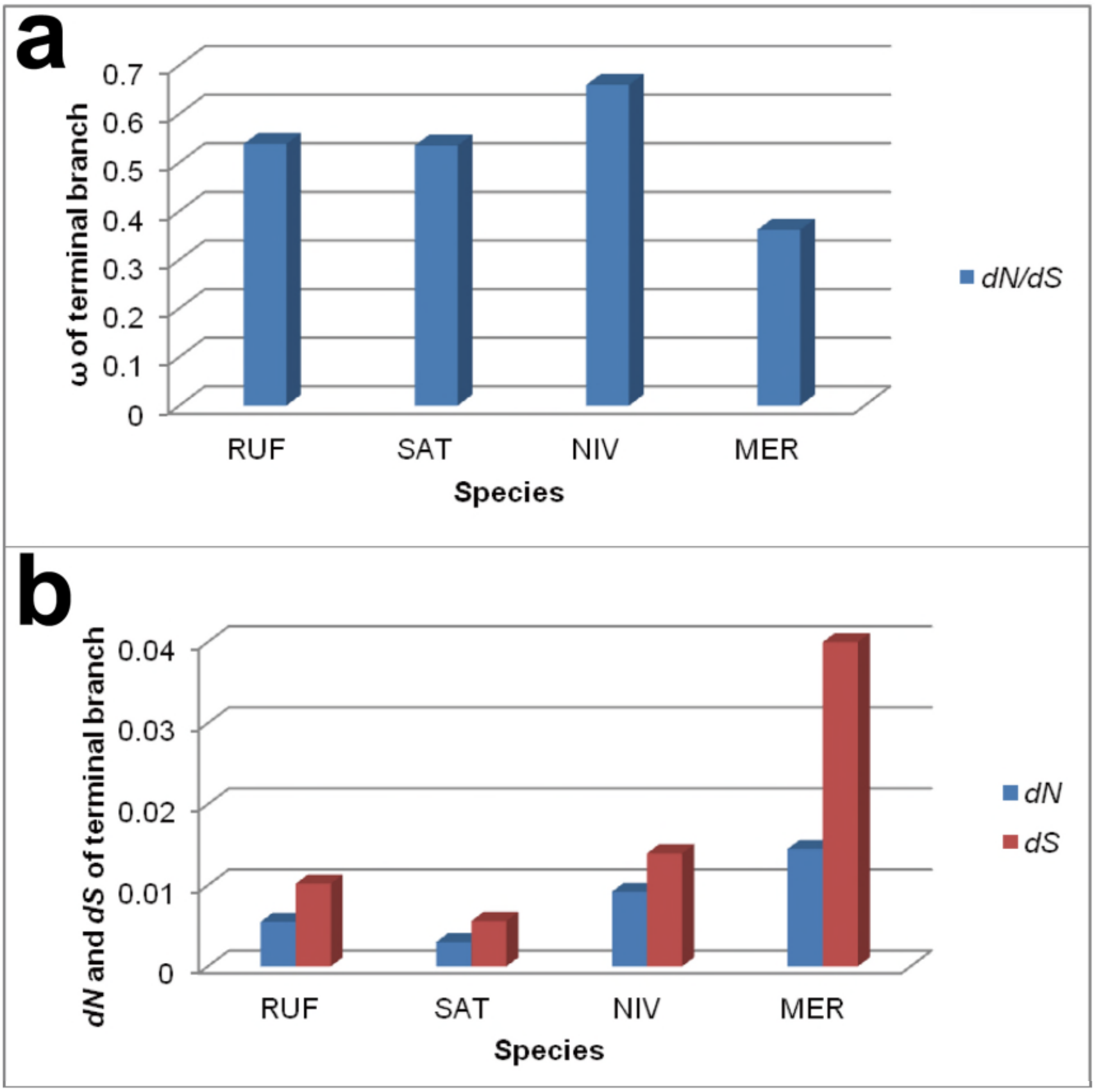

Supplementary Figure 10. Branch-specific *dN*, *dS* and ω values in each terminal branch. Shown are values of **a** ω (*dN*/*dS*); **b** *dN* and *dS* for each species, which were estimated in branch model tests.

Supplementary Tables

Supplementary Table 1. Assembly statistics of the *O. rufipogon* genome.

	Assembler			
	**Falcon-Unzip**	**Falcon-Unzip+10×**	**Falcon-Unzip+ 10×+Hi-C**	**Falcon-Unzip+10 ×+Hi-C+PBJelly**
**Assembled Length (bp)**	373,883,792	377,175,457	373,971,792	380,512,861
**Scaffold N50 (bp)**	710,332	2,214,952	29,494,264	30,197,710
**Scaffold N90 (bp)**	178,741	487,719	22,265,879	22,824,363
**Contig N50 (bp)**	710,332	710,332	710,332	1,096,430
**Contig N90 (bp)**	178,741	178,741	178,741	286,860
**Scaffold Number**	1,404	843	524	524
**Contig Number**	1,404	1,404	1,404	1,077
**Longest Scaffold (bp)**	4,290,542	7,550,243	43,695,925	44,260,698
**Longest Contig (bp)**	4,290,542	4,290,542	4,290,542	4,409,078

Supplementary Table 2. Assembly results of p-contigs and haplotigs of the *O. rufipogon* genome..

**Sequences**	**Assembly length (Mb)**	**No. contigs**	**N50 length (Kb)**	**N90 length (Kb)**	**Max contig length (Kb)**
**P-contigs**	373.88	1,404	710.33	178.74	4,290.54
**Haplotigs**	23.85	843	29.47	18.51	653.91

Supplementary Table 3. Scaffold length constitutions of *O. rufipogon*.

Scaffold Length	Number	Scaffold Length (bp)	Average Length (bp)	Percentage (%)
**>1 Kb**	826	377,165,817	456,617	100.00
**>10 Kb**	600	375,914,405	626,524	99.67
**>50 Kb**	331	370,046,179	1,117,964	98.11
**>100 Kb**	290	367,168,813	1,266,099	97.35
**>200 Kb**	252	361,676,028	1,435,222	95.89
**>300 Kb**	230	356,507,333	1,550,031	94.52
**>500 Kb**	184	337,877,604	1,836,291	89.58
**>800 Kb**	141	311,420,982	2,208,659	82.57
**>1 Mb**	122	294,593,993	2,414,705	78.11
**>2 Mb**	59	204,848,868	3,472,015	54.31
**>3 Mb**	29	132,571,336	4,571,425	35.15

Supplementary Table 4. Assembly statistics for the *O. rufipogon* genome sequences.

Chromosome ID	Chromosome Length (RUF) (bp)	Chromosome length (Nipponbare) (bp)
1	43,695,925	43,270,923
2	39,958,125	35,937,250
3	37,088,850	36,413,819
4	29,494,264	35,502,694
5	28,993,391	29,958,434
6	31,585,856	31,248,787
7	26,272,893	29,697,621
8	27,299,189	28,443,022
9	21,773,623	23,012,720
10	26,444,676	23,207,287
11	29,583,747	29,021,106
12	22,265,879	27,531,856
Unmapped	9,515,374	0
Total	373,971,792	373,245,519

Supplementary Table 5. Statistics for large variants detected between haplotigs and p-contigs.

Variant Type	Count	Total length (bp)
**Insertion**	429	21,114
**Deletion**	247	22,989
**Tandem_expansion**	16	85,296
**Tandem_contraction**	2	5,617
**Repeat_expansion**	9	32,690
**Repeat_contraction**	1	4,301
**Total**	704	172,007

Supplementary Table 6. Quality assessment of the *O. rufipogon* genome assembly using reads mapping, DNA, protein and EST datasets.

	Total	Aligned	Percentage (%)
**Reads mapping**			
PE reads	175,474,936	163,187,767	93.00
**Sequences available in public databases**			
DNA *	20,667	17,968	86.94
Protein **	3,762	2,424	64.43
**Transcripts of *O. rufipogon***			
EST *	105,654	75,374	71.34
**BUSCOs from Embryophyta lineage*** **			
Complete	1,440	1,402	97.36
Duplicated	1,440	17	1.18
Fragmented	1,440	11	0.76
Missing	1,440	27	1.88

*Aligned using GMAP (version 2014-10-22), and the hits with identity ≥ 90% && coverage ≥ 90% are retained.

**Aligned using genBlastA (version 1.0.1), and the hits with identity ≥ 80% && coverage ≥ 90% are retained.

***The 1,440 BUSCO conserved genes used were collected from Embryophyta lineage.

Supplementary Table 7. Summary of gene prediction for *O. rufipogon*.

Type		Values
**Gene Features**	Total number of predicted genes (#)	34,830
	Total number of gene models (#)	34,830
	Average gene length (bp)	2,921
	Average CDS length (bp)	1,125
	Average exons per gene	4.5
	Average exon length (bp)	248
	Average intron length (bp)	507

Supplementary Table 8. Validation of gene models of *O. rufipogon* by using homologous proteins and RNA-Seq datasets.

Evidence	Resources	Number	% Percent
Total predicted genes	This study	34,589	100
Homologous protein supported*	*O. sativa*	24,711	71.44
RNA-Seq supported**	This study	21,515	62.20
Homology protein or RNA-Seq supported		29,123	84.20

Note: Blast with e-value < $1e^{-5}$ was used for searching against database;

* Identity >= 30% and Coverage >= 80%;

** Identity >= 90% and Coverage >= 50%

Supplementary Table 9. Summary of non-coding RNA genes in the *O. rufipogon*.

Type	Number	Average Length (bp)	Total Length (bp)	% of Genome
tRNA	637	75	47,862	0.0127
rRNA (8S)	976	116	113,091	0.0300
rRNA (18S)	56	1,814	101,573	0.0269
rRNA (28S)	53	3,933	208,438	0.0553
SnoRNA	442	120	53,026	0.0141
snRNA	117	143	16,713	0.0044
miRNA	245	140	34,236	0.0091

Supplementary Table 10. Statistics of repeat sequence in the *O. rufipogon* genome.

	**Length (bp)**	**Percentage (%)**
**Transposable Elements**	166467134	44.14
**DNA transposons**	56904501	15.09
En-Spm	13033370	3.46
Harbinger	5602726	1.49
MuDR	15643183	4.15
TcMar-Stowaway	3271049	0.87
Tourist	14,711	0.00
hAT	3900448	1.03
Helitron	11628837	3.08
**Other**	3810177	1.01
**Retrotransposon**	102758700	27.24
**Non-LTR Retrotransposon**	5174466	1.37
**LINE**	4498862	1.19
**SINE**	675604	0.18
**LTR Retrotransposon**	97584234	25.87
Copia	11212454	2.97
Gypsy	58639158	15.55
**Other***	27732622	7.35
**Other Repeats**	6803933	1.80
**Satellite**	1168	0.00
**Simple repeats**	4799297	1.27
**Unknown**	2003468	0.53

* Non-autonomous LTR retrotransposons.

Supplementary Table 11. Occurrence of simple sequence repeats (SSRs) in the *O. rufipogon* genome.

Repeat type	Number	Proportion (%)	Total length (Kb)	Average length (bp)
Mononucleotide	16,790	7.67	238.82	14
Dinucleotide	39,838	18.19	964.59	24
Trinucleotide	84,516	38.60	1,190.43	14
Tetranucleotide	49,983	22.83	659.63	13
Pentanucleotide	16,967	7.75	271.29	16
Hexanucleotide	10,873	4.97	208.58	19
**Total**	218,967	100.00	3,533.34	16

Supplementary Table 12. Assembly statistics of *O. rufipogon*, *O.sativa* and *O. nivara* genome.

	O.sativa	***O. nivara***	***O. rufipogon***
**Sequencing technology**	Sanger	Illumina	PacBio
**Completeness (BUSCO) (%)**	98.2	95.4	97.36
**Contig N50 (bp)**	7,711,345	13,868	1,096,430
**Scaffold N50 (bp)**	29,958,434	506,802	30,197,710
**Total length (bp)**	374,471,240	379,501,719	380,512,861
**Protein No.**	39,045	41,490	34,830

Supplementary Table 13. Summary of the pan-genome of *O. rufipogon*, *O. sativa* and *O. nivara*.

	Sequence length (bp)
Shared among RUF, NIV and SAT	317,729,226
Shared between RUF and NIV	349,534,715
Shared between RUF and SAT	342,883,315
Shared between NIV and SAT	336,486,012
RUF-specific	98,013,406
NIV-specific	11,210,218
SAT-specific	12,831,139
Pan-genome	515,500,353

Note: *O. sativa* is abbreviated as SAT, *O. rufipogon* is abbreviated as RUF and *O. nivara* is abbreviated as NIV.

Supplementary Table 14. Enrichment of core and dispensable genes in various GO categories.

	GO term	Frequency in core genes	Frequency in dispensable genes	*P*-value	FDR
**Core genes**					
BP	Biological regulation	0.0988	0.0747	2.19E-06	2.63E-05
BP	Regulation of biological process	0.0970	0.0739	5.05E-06	3.03E-05
CP	Cell	0.1165	0.0923	1.13E-05	4.51E-05
CP	Cell part	0.1165	0.0923	1.13E-05	3.38E-05
BP	Cellular component organization or biogenesis	0.0261	0.0166	2.57E-04	6.18E-04
BP	Localization	0.0859	0.0699	7.08E-04	1.42E-03
BP	Cellular process	0.3798	0.3527	1.21E-03	2.08E-03
BP	Single-organism process	0.2467	0.2230	1.31E-03	1.97E-03
CP	Organelle	0.0784	0.0646	1.95E-03	2.60E-03
MF	Nucleic acid binding transcription factora ctivity	0.0353	0.0267	4.23E-03	5.07E-03
CP	Membrane part	0.0675	0.0596	4.11E-02	4.48E-02
MF	Protein binding transcription factor activity	0.0036	0.0020	4.86E-02	4.86E-02
**Dispensable genes**					
BP	Reproductive process	0.0026	0.0123	9.26E-11	9.26E-10
BP	Multi-organism process	0.0031	0.0127	3.69E-10	1.85E-09
BP	Multicellular organismal process	0.0054	0.0150	3.91E-08	1.30E-07
MF	Nutrient reservoir activity	0.0025	0.0073	4.98E-05	1.24E-04
MF	Structural molecule activity	0.0136	0.0216	4.01E-04	8.03E-04
MF	Catalytic activity	0.4345	0.4578	5.62E-03	9.37E-03
BP	Growth	0.0004	0.0016	2.09E-02	2.98E-02
MF	Enzyme regulator activity	0.0090	0.0123	4.27E-02	5.34E-02
MF	Electron carrier activity	0.0087	0.0119	4.53E-02	5.03E-02
MF	Metallochaperone activity	0.0000	0.0004	4.96E-02	4.96E-02

Supplementary Table 15. Summary of genomic variation among *O. sativa*, *O. rufipogon* and *O. nivara*.

A. Combined method of genome comparison and reads mapping analysis

**Species**	**Genomic Variation**			
	Number of SNP	Number of InDel	Number of SV	Number of CNV
RUF	4,997,466	817,238	16,163	5,017
NIV	3,794,980	779,252	19,914	7,766
SAT	23,584	2,764	1,879	2,034

Note: Numbers of SNP and InDel were obtained from whole genome comparisons and reads mapping analysis; SV and CNV calling was only based on reads mapping analysis.

B. Genome comparison method

**Species**	**Genomic Variation**	
	Number of SNP	Number of InDel
RUF	2,887,048	551,018
NIV	3,093,758	619,797

Note: SNP and InDel calling are based on whole genome alignments.

C. Reads mapping method

**Species**	**Genomic Variation**			
	Number of SNP	Number of InDel	Number of SV	Number of CNV
RUF	4,065,179	403,544	16,163	5,017
NIV	2,841,519	319,771	19,914	7,766
SAT	23,584	2,764	1,879	2,034

Note: *O. sativa* is abbreviated as SAT, *O. rufipogon* is abbreviated as RUF and *O. nivara* is abbreviated as NIV.

Supplementary Table 16. Annotation of genomic variation among *O. sativa*, *O. rufipogon* and *O. nivara*.

A. SNP annotation

**Species**	**Number of SNP**			
	Number of Synonymous Sites	Number of Non-synonymous Sites	Number of Stop codon Gain	Number of Stop codon Loss
RUF	339,831	446,309	17,124	2,218
NIV	238,642	349,519	14,083	1,730

B. InDel annotation

**Species**	**Number of InDel**			
	Number of Stop codon Loss	Number of Stop codon Gain	Number of FrameShift	Number of Non-FrameShift
RUF	109	920	25,139	14,614
NIV	88	1,242	41,038	13,568

C. CNV annotation

**Species**	**Number of CNV**	
	Number of Deletion	Number of Duplication
RUF	3,355	1,662
NIV	3,562	4,204
SAT	1,806	228

Note: Deletion and duplication refer that copy number fewer and more than in the reference genome, respectively.

D. SV annotation

**Species**	**SV**						
	Number of ICT1	Number of Deletion	Number of Insertion	Number of Inversion	Number of ITC2	Number of Exon	
						Number of FrameShift	Number of Non-FrameShift
RUF	52	12,035	0	465	621	4,539	266
NIV	185	13,655	2,524	384	431	4,660	554
SAT	3	47	1,357	26	20	228	91

Note: ICT1 refers to Intra-chromosome translocation; ITX2 refers to Inter-chromosome translocation. SVs that occur within the exon regions were classified them into two types, frameshift and non-frameshift. *O. sativa* is abbreviated as SAT, *O. rufipogon* is abbreviated as RUF and *O. nivara* is abbreviated as NIV.

Supplementary Table 17. Shared and sample-specific genes affected by CNVs in the *O. rufipogon* and *O. nivara* genomes.

	Number of shared genes	
	N=2	N=1
**Loss**	88	940
**Gain**	145	6,940
**Loss/Gain**	86	-
**Total**	319	7,880

Supplementary Table 18. Numbers of MADS-box genes affected by CNVs and total MADS-box genes from whole genome of *O. sativa*.

**MADS-box type**	**Affected by CNVs**	**Whole genome**
M-α	4	13
M-β	2	9
M-γ	0	10
**Type I (subtotal)**	6	32
$MIKC^C$	15	37
MIKC*	2	5
**Type II (subtotal)**	17	42
**Total**	23	74

Supplementary Table 19. The 23 MADS-box genes affected by CNVs in *O. sativa*.

Gene ID	Physical location	Type	#Wild species	MADS-box type
LOC_Os01g10504	5559548-5568844	CNV-gain	1	MIKCc
LOC_Os01g66290	38500880-38505127	CNV-gain	1	MIKCc
LOC_Os01g67890	39459599-39461050	CNV-gain	1	M-beta
LOC_Os01g69850	40344329-40364584	CNV-gain	1	MIKC*
LOC_Os02g07430	3833129-3837135	CNV-gain	1	MIKCc
LOC_Os02g36924	22294657-22301808	CNV-gain	1	MIKCc
LOC_Os02g52340	32038902-32045130	CNV-gain	1	MIKCc
LOC_Os03g11614	6052750-6061369	CNV-gain	1	MIKCc
LOC_Os04g23910	13672710-13675884	CNV-gain	1	MIKCc
LOC_Os04g31804	19042703-19058654	CNV-gain	1	M-alpha
LOC_Os04g52410	31143083-31144886	CNV-gain	1	MIKCc
LOC_Os05g23780	13656346-13657002	CNV-gain	1	M-alpha
LOC_Os06g06750	3162801-3169415	CNV-gain	1	MIKCc
LOC_Os06g45650	27637555-27641578	CNV-gain	1	MIKCc
LOC_Os07g04170	1781625-1785426	CNV-gain	1	M-beta
LOC_Os07g41370	24788476-24793884	CNV-gain	1	MIKCc
LOC_Os08g02070	679358-681739	CNV-gain	1	MIKCc
LOC_Os08g20440	12272625-12277708	CNV-gain	1	M-alpha
LOC_Os08g33488	20897215-20906881	CNV-gain	1	MIKCc
LOC_Os08g41950	26507180-26512261	CNV-gain	1	MIKCc
LOC_Os11g43740	26414394-26418442	CNV-gain	1	MIKC*
LOC_Os12g10540	5584593-5590285	CNV-gain	1	MIKCc
LOC_Os12g21850	12303478-12304062	CNV-gain	1	M-alpha

Supplementary Table 20. Numbers of flowering-related genes affected by CNVs and total flowering-related genes from whole genome of *O. sativa*.

Flowering-related genes	Affected by CNVs	Whole genome
CAB	4	14
CCA	5	18
CDF	0	5
CO	1	7
COP	1	1
CRY	1	6
ELF	0	3
FKF	0	1
FT	4	18
GI	1	1
LFY	0	1
LHY	0	1
Lux	6	29
PHOT	6	27
PHY	2	3
PIF_PIL	0	2
PRR	3	19
SOC	17	53
TOC	0	1
ZTL	2	4
**Total**	**53**	**210**

* Flowering-related genes in rice were detected using BLASTP (Identity ≥ 30% && Coverage ≥ 70%) based on the homologous protein sequences from *Arabidopsis thaliana*.

Supplementary Table 21. Rice flower development pathway-associated genes affected by CNVs.

GeneID	Physical location	Type	Function	Symbol
LOC_Os01g07790	3737893-3740685	CNV-gain	Polygalacturonase, putative, expressed	*PG*
LOC_Os01g23740	13353629-13357510	CNV-gain	OsPDIL2-2 protein disulfide isomerase PDIL2-2, expressed	*PDIL*
LOC_Os02g34850	20899808-20907175	CNV-gain	Histone-lysine N-methyltransferase ASHH2, putative, expressed	*ASHH2*
LOC_Os03g18200	10204819-10210306	CNV-gain	Heat shock protein DnaJ, putative, expressed	*TMS1*
LOC_Os04g34450	20862474-20872470	CNV-gain	Expressed protein	*SEC5*
LOC_Os04g49450	29500951-29503775	CNV-gain	MYB family transcription factor, putative, expressed	*LHY*
LOC_Os05g05310	2611683-2617251	CNV-gain	Fibronectin type III domain containing protein, expressed	*VIL1*
LOC_Os07g06970	3429443-3434747	CNV-gain	HEN1, putative, expressed	*HEN1*
LOC_Os09g13610	7914083-7925405	CNV-gain	PFT1, putative, expressed	*PFT1*
LOC_Os10g02770	1092789-1096966	CNV-gain	Glycosyl hydrolases family 16, putative, expressed	*XTH*
LOC_Os10g27470	14487133-14493136	CNV-gain	KH domain containing protein, putative, expressed	*PEP*

Supplementary Table 22. Number of *R*-genes affected by CNVs and total *R*-genes from whole genome of *O. sativa*.

**Type**	**Located in CNV region**	**Whole genome**
CC-NBS	8	30
CC-NBS-LRR	34	73
NBS-LRR	96	322
TIR-NBS	1	1
NBS only	57	186
Total	196	612

Supplementary Table 23. Summary of PAV calling between *O. sativa* and *O. rufipogon*, *O. sativa* and *O. nivara*, respectively. PAV types are represented in a customized format, of which RS10 indicates that a PAV is present RUF but absent in SAT, NS10 indicates that a PAV is present NIV but absent in SAT, RS01 indicates that a PAV is absent RUF but present in SAT, and NS01 indicates a PAV is absent in NIV but present in SAT.

**PAV**	**Number of PAVs**						
**Type**	100-500	501-1000	1001-2000	2001-5000	5001-20000	>20000	**Total**
**RS10**	11,255	5,310	4,362	3,236	1,198	26	**25,387**
**RS01**	5,773	1,845	1,148	1,143	599	11	**10,519**
**NS10**	6,448	2,888	2,431	1,962	702	8	**14,439**
**NS01**	19,735	7,477	4,192	2,670	1,083	24	**35,181**

Supplementary Table 24. Summary of PAV calling between *O. sativa* and *O. rufipogon*, *O. sativa* and *O. nivara*, respectively.

PAV Type	Length of PAV regions (bp)	Gene number in PAV regions	Gene number with known functions
**RS10**	16,232,326	4,621	3,018
**RS01**	9,503,214	3,241	1,123
**NS10**	9,161,166	3,076	1,701
**NS01**	23,220,789	14,425	6,906

Note: R: *O. rufipogon*; S: *O. sativa*; N: *O. nivara*; 1: presence; 0: absence.

Supplementary Table 25. Clustering of gene families among the three *Oryza* species, *O. sativa*, *O. rufipogon* and *O. nivara*.

**Species**	**Gene number**	**Genes in families**	**Unclustered genes**	**Family number**	**Unique families**	**Average gene number per family**
**RUF**	48,445	36,902	11,543	25,391	1,007	1.45
**NIV**	38,881	29,911	8,970	24,095	437	1.24
**SAT**	39,043	33,425	5,618	26,043	239	1.28

Supplementary Table 26. Number of putative NBS-LRR genes identified in the three *Oryza* species, *O. sativa*, *O. rufipogon* and *O. nivara*.

	**RUF**	**SAT**	**NIV**
CC-NBS	76	56	64
CC-NBS-LRR	166	252	133
NBS-LRR	215	227	178
TIR-NBS	1	1	1
NBS	118	95	113
Total	**576**	**631**	**489**

Supplementary Table 27. Summary of branch-specific ω, *dN*, and *dS* values along *O. rufipogon*, *O. sativa*, *O. nivara* and *O. meridionalis* lineages estimated by using PAML.

Species/branch[a]	ω(*dN*/*dS*)	*dN*	*dS*
**1# RUF**	0.5382	0.005491	0.010202
**2# SAT**	0.5352	0.002995	0.005596
**3# NIV**	0.6598	0.009203	0.013947
**4# MER**	0.3618	0.014485	0.040033
**5# Ancestral of RUF&SAT**	0.6333	0.001826	0.002883

Supplementary Table 28. Summary of the numbers of PSGs detected along *O. rufipogon*, *O. sativa* and *O. nivara* lineages estimated by using PAML.

Branch	All PSGs		Lineage-specific PSGs [c]	
	P < 0.05	FDR < 0.05	P < 0.05	FDR < 0.05
[a] All branches	1,905	1,799	-	-
SAT	273	247	103	90
NIV	1,017	996	487	476
RUF	440	416	211	199
[b] Ancestral of RUF&SAT	250	235	53	50
Total (non-redundancy)	2,148	2,053	854	815

[a] The genes under selection along any branch of the phylogenetic tree based on the site model;

[b] The branch leading to Asian cultivated rice and *O. rufipogon*;

[c] These genes show significant evidence of positive selection only along one branch.
