# Supplementary material for: SMRT sequencing of the Oryza rufipogon genome reveals the genomic basis of rice adaptation
Source: Commun Biol. 2020 Apr 7;3:167. doi: 10.1038/s42003-020-0890-8 (PMC7138787; doi:10.1038/s42003-020-0890-8)
Supplement: Supplementary file 13 — Description of Additional Supplementary Files [file 42003_2020_890_MOESM13_ESM.pdf]

Description of Additional Supplementary Files

Supplementary Data 1. List of the identified genes affected by CNVs between the RUF/NIV and SAT genomes.

Supplementary Data 2. GO and PFAM functional enrichment analysis of genes affected by CNVs in the RUF and NIV genomes.

Supplementary Data 3. The 53 flowering-related genes affected by CNVs in *O. sativa*.

Supplementary Data 4. The 196 R-genes affected by CNVs in *O. sativa*. * Number of wild species.

Supplementary Data 5. PFAM and GO functional enrichment analysis of genes associated with PAVs. The top 10 Pfam and GO IDs are given for each PAV type. Significance levels of functional enrichment are shown for P-value and FDR.

Supplementary Data 6. Gene Ontology(GO) and PFAM functional enrichment anlysis of RUF, NIV and SAT-specific genes.

Supplementary Data 7. Summary of lineage-specific gene families identified among *O. sativa*, *O. rufipogon*, *O. nivara* and *O. meridionalis*.

Supplementary Data 8. Comparison of functional enrichment of expanded and contracted gene families in the RUF, NIV and SAT genomes.

Supplementary Data 9. List of positively selected genes. **a** List of positively selected genes in the three AA-genome *Oryza* species based on site-model. **b** List of positively selected genes in the three AA-genome *Oryza* species based on branch-site model (SAT). **c** List of positively selected genes in the three AA-genome *Oryza* species based on branch-site model (RUF). **d** List of positively selected genes in the three AA-genome *Oryza* species based on branch-site model (NIV). **e** List of positively selected genes in the three AA-genome *Oryza* species based on branch-site model (ancestral of RUF && SAT). **f** List of positively selected genes in the three AA-genome *Oryza* species based on (branch-site model: clade of RUF && SAT).

Supplementary Data 10. Functional enrichment analysis of the detected PSGs along *O. rufipogon*, *O. sativa* and *O. nivara* lineages.

Supplementary Data 11. Summary of the detected PSGs orthologous to important functions in *O. rufipogon*, *O. sativa* and *O. nivara*.
